# Photoelastic Stress Field Recovery Using Deep Convolutional Neural Network

**DOI:** 10.3389/fbioe.2022.818112

**Published:** 2022-03-21

**Authors:** Bo Tao, Yan Wang, Xinbo Qian, Xiliang Tong, Fuqiang He, Weiping Yao, Bin Chen, Baojia Chen

**Affiliations:** ^1^ Key Laboratory of Metallurgical Equipment and Control Technology, Ministry of Education, Wuhan University of Science and Technology, Wuhan, China; ^2^ Hubei Key Laboratory of Mechanical Transmission and Manufacturing Engineering, Wuhan University of Science and Technology, Wuhan, China; ^3^ Precision Manufacturing Institute, Wuhan University of Science and Technology, Wuhan, China; ^4^ Research Center for Biomimetic Robot and Intelligent Measurement and Control, Wuhan University of Science and Technology, Wuhan, China; ^5^ Hubei Key Laboratory of Hydroelectric Machinery Design and Maintenance, China Three Gorges University, CTGU, Yichang, China

**Keywords:** neural network, deep learning, inverse problem solving, stress evaluation, digital photoelasticity

## Abstract

Recent work has shown that deep convolutional neural network is capable of solving inverse problems in computational imaging, and recovering the stress field of the loaded object from the photoelastic fringe pattern can also be regarded as an inverse problem solving process. However, the formation of the fringe pattern is affected by the geometry of the specimen and experimental configuration. When the loaded object produces complex fringe distribution, the traditional stress analysis methods still face difficulty in unwrapping. In this study, a deep convolutional neural network based on the encoder–decoder structure is proposed, which can accurately decode stress distribution information from complex photoelastic fringe images generated under different experimental configurations. The proposed method is validated on a synthetic dataset, and the quality of stress distribution images generated by the network model is evaluated using mean squared error (MSE), structural similarity index measure (SSIM), peak signal-to-noise ratio (PSNR), and other evaluation indexes. The results show that the proposed stress recovery network can achieve an average performance of more than 0.99 on the SSIM.

## 1 Introduction

Inspired by the human nervous system, Rosenblatt (1958) proposed the perceptron model, which became the basis of the early artificial neural network (ANN). In recent years, the ANN, especially deep neural network (DNN), has become one of the fastest developing and most widely used artificial intelligence technologies(Sun et al., 2020a; Sun et al., 2020c; Li et al., 2020; Tan et al., 2020; Chen et al., 2021a). Classical research work has proved the excellent performance of DNNs in image classification (He et al., 2016), medical image segmentation (Ronneberger et al., 2015; Xiu Li et al., 2019; Jiang et al., 2021a), image generation (Isola et al., 2017), and depth estimation (Godard et al., 2017; Jiang et al., 2019a). The deep convolutional neural network is widely used in feature extraction of image data (Jiang et al., 2019b; Huang et al., 2020; Hao et al., 2021). As an extremely powerful tool, the deep convolutional neural network can provide a new perspective for the application of digital photoelasticity. That is, the deep convolutional neural network can directly learn the corresponding relationship between isochromatic pattern and principal stress difference pattern.

In digital photoelasticity, the fringe patterns that contain the whole field stress information in terms of the difference of principal stresses (isochromatics) and their orientation (isoclinics) are captured as a digital image, which is processed for quantitative evaluation (Ramesh and Sasikumar, 2020). Frequency-domain and spatial-domain analysis methods, such as Fourier transform (Ramesh et al., 2011), phase-shift (Tao et al., 2022), step-loading method (Ng, 1997; Zhao et al., 2022) and multiwavelength technology (Dong et al., 2018), are usually used to process the data of isochromatics and isoclinics. However, in the actual industrial scene, the traditional pattern demodulation will face challenges, such as the color difference in the color matching method of the calibration table; the complex geometry of the sample, which makes the pattern complex and difficult to demodulate; and the fringes with different experimental configuration need special analysis, all of which make photoelasticity research a complex process extending to industrial applications.

In recent years, deep learning has shown increasing interest in solving traditional mechanics problems (Chen et al., 2021b; Jiang et al., 2021b; Duan et al., 2021; Tao et al., 2021; Zhang et al., 2022), which is due to deep learning’s powerful ability of data feature extraction and representation of complex relationships. In general, deep learning is committed to mining implicit rules of data from a large number of data sets and then using the learned rules to predict the results and hoping that the learned models have good generalization ability (Cheng et al., 2021; Huang et al., 2021; Yang et al., 2021; Chen et al., 2022). Works in optical image processing, such as phase imaging (Gongfa Li et al., 2019; Xin Liu et al., 2021; Sun et al., 2022), phase unwrapping (Wang et al., 2019), and fringe pattern analysis (Feng et al., 2019), have also demonstrated the applicability of deep learning. Recovering the full-field principal stress difference of the loaded object from the photoelastic fringe pattern can be regarded as an inverse problem solving process of deep learning. A large number of datasets are collected and trained to find the complex correspondence between the fringe pattern and stress difference pattern, which is then used to recover the stress field of the real loaded object from a single fringe pattern. When many conditions such as specimen shape, material properties, and the setting of polarized light field need to be considered, it is difficult for traditional mathematical methods to deal with this complicated and changeable situation. However, with sufficient data collected under different experimental conditions, deep learning can directly learn the complex correspondence between the input fringe pattern and output principal stress difference.

In this study, a deep convolutional neural network model is designed for inferring the stress field from the photoelastic fringe pattern. The overall framework of the network is in the form of encoder–decoder structure. The encoder completes the feature extraction process of the input fringe pattern, and the decoder completes the process of feature fusion to stress distribution pattern inference, thus realizing the transformation from the single photoelastic fringe pattern to stress distribution pattern. The main contributions of our study can be summarized as follows:

(1) A simple and efficient stress recovery neural network is designed to realize the process of stress field recovery of the loaded object from a single fringe pattern.
(2) A multiloss function weighted objective optimization function is proposed to accelerate the convergence of neural networks and improve the robustness of model prediction.
(3) The superior performance of the proposed method is verified on a public dataset.

The remainder of this article is structured as follows. Some of the work closely related to this study will be discussed in Section 2. The combination of the photoelastic method and convolutional neural network and the design of the neural network model and objective optimization function is presented in Section 3. In Section 4, the details of the experiment implementation are introduced, the method proposed in this study is compared with that of other studies in detail, and then the experimental results are further analyzed. Finally, the conclusion and limitations of the proposed method are given in Section 5.

## 2 Related Work

It is a challenging task to recover the stress field of the loaded object from a single photoelastic fringe pattern. Most of the traditional methods are limited by different experimental conditions and calculation methods when dealing with complex fringe patterns. Recently reported methods based on deep learning provide new ideas to solve these shortcomings. Feng et al. (2019) proposed a fringe pattern analysis method based on deep learning. They collected phase-shifted fringe patterns in different scenes to generate training data and then trained neural networks to predict some intermediate results. Finally, combining these intermediate results, the high-precision phase image is recovered by using arc tangent function. The results show that this method can significantly improve the quality of phase recovery. Sergazinov and Kramar (2021) use the CNN to solve the problem of force reconstruction of photoelastic materials. They use the synthetic dataset obtained by theoretical calculation for training and then use the transfer learning to fine-tune a small amount of real experimental data, which shows good force reconstruction results.

For the estimation of the photoelastic stress field under a single experimental condition, a dynamic photoelastic experimental method based on pattern recognition was proposed (Briñez-de León et al., 2020a). The ANN was used to process the color fringe patterns that changed with time so as to classify the stress of different sizes, isotropic points, and inconsistent information. In order to make the deep learning method suitable for a wider range of experimental conditions, Briñez-de León et al. (2020b) reported a powerful synthetic dataset which covered photoelastic fringe patterns and the corresponding stress field distribution patterns under various experimental conditions with highly diversified spatial fringe distribution. At the same time, a neural network structure based on VGG16 (Simonyan and Zisserman, 2014) is proposed to recover the stress field from the isochromatic pattern. However, the prediction results of this network model are somewhat different from the real maximum stress difference, and the prediction results of the stress on the rounded surfaces are not very accurate. In their reports in the other literature (Briñez-de León et al., 2020c), an image translation problem directly related to spatial transformation based on the generative adversarial network (GAN) model was proposed. This method showed good performance in the SSIM, but there is a supersaturation phenomenon of stress recovery in some specimens. In addition, GAN is not an easy training model for the convergence of the network (Sun et al., 2021; Ying Liu et al., 2021; Wu et al., 2022). Recently, they proposed a new neural network model to evaluate the stress field and named it PhotoelastNet (Briñez-de León et al., 2022). Considering the influence of noise and complex stress distribution patterns, the scale of synthetic data was further expanded, and a lighter network structure was designed, which achieved better performance in synthetic images and experimental images. However, there is still a certain gap between the accuracy of stress distribution estimation and ground truth. Our study improves on these methods by proposing a simpler and reasonable network structure and designing more effective loss functions to solve these problems.

## 3 Photoelasticity and the Neural Network Model

### 3.1 Photoelasticity

**FIGURE 1:**
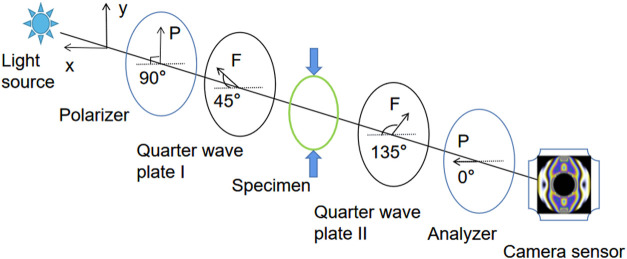
Schematic diagram of the orthogonal circularly polarized light field.

Photoelastic fringes are visualized patterns obtained by a polarized optical system, which display the invisible stress response of each point in the model related to the birefringence effect through the related optical system. In this research, photoelastic fringes are visualized by a circular polariscope. The schematic diagram of the circular polariscope is shown in Figure 1. The circular polariscope comprises a light source, polarizer, quarter wave plate I (QW-I), specimen which is made of photoelastic material, quarter wave plate II (QW-II), and an analyzer.

In Figure 1, *F* represents the fast axis of the QW-I and QW-II. The orientations of the fast axis of the QW-I and QW-II are set to 45° and 135°, respectively. The orientation of the polarizer is set to 90°. The orientation of the analyzer is set to 0°. The light carried with the specimen’s stress information is emitted from the analyzer, and the light intensity can be expressed as follows:

$$I_{a}=I_{b}+\frac{I_{0}}{2}\left(1-\cos\delta \right),$$ (1)

where *I*_b_ is the background light intensity. I_0_ is the intensity of the light source. *δ* is the isochromatic angle of the photoelastic model, which contains the stress field information.

According to the law of stress optics, the principal stress difference in photoelastic models is proportional to the refractive index in the principal stress direction, as shown in Eq. 2. The optical path difference produced when polarized light passes through the photoelastic model can be expressed as Eq. 3.

$$n_{1}-n_{2}=C\left(\sigma_{1}-\sigma_{2}\right),$$ (2)

$$\text{z}=\left({\text{n}}_{1}-{\text{n}}_{2}\right)\text{h}=\text{λδ}/2\text{π},$$ (3)

where *σ*_i_ is the ith principal stress, *n*_i_ is the refractive index in the direction of *σ*_i_, *C* = *C*_1_ − *C*_2_, *C*_*i*_ is the optical coefficient of the stress of the model material, *δ* is the phase delay, *h* is the thickness of the photoelastic model, z is the optical path difference, and *λ* is the wavelength of the incident light source.

In two-dimensional photoelasticity, the relationship between phase delay caused by principal stress difference and the properties of the optical material is shown in Eq. 4. The main influencing factors of phase delay are light wavelength, optical coefficient of photoelastic material, model thickness, and stress condition.

$$\delta =2\pi \left(\sigma_{1}-\sigma_{2}\right)h/f_{\sigma },$$ (4)

where *f*_σ_ = *λ*/*C* is the material fringe value.

The photoelastic fringe patterns collected by the camera are interference intensity images of light. These fringe patterns generated by phase delay wrap the stress field information of the stressed object. In general, the stress field can be understood as the mechanical effect caused by the force distributed inside the object (Markides and Kourkoulis, 2012), which can be expressed by the principal stress difference σ_1_ − σ_2_.

Briñez-de León et al. (2020b) reported that the intensity of the emitted light is related to the spectral content of the light source, optical elements in the polarized light system, spatial stress distribution, and relative spectral response in the camera sensor. Based on the circular polariscope shown in Figure 1, the relationship between the intensity and phase delay of the emitted light (Ajovalasit et al., 2015) in different color channels is shown in Eq. (5).

$$I_{R,G,B}=\frac{1}{\lambda_{2}-\lambda_{1}}\int_{\lambda_{1}}^{\lambda_{2}}\frac{I_{0}\left(\lambda \right)}{2}\left[1-\cos\delta \left(\lambda \right)\right]S_{R,G,B}\left(\lambda \right)d\lambda ,$$ (5)

where *I* is the emergent light intensity; RGB is the red, green, and blue color channels; and $S_{R,G,B}\left(\lambda \right)$ is the relative spectral response of the camera.

According to the causal relationship between the photoelastic fringe pattern and stress field, the process from the stress field to fringe pattern can be regarded as a forward problem, while solving the stress field according to the single fringe pattern is a challenging inverse problem, that is, unwrapping stress information in the fringe pattern. We propose a stress field recovering method based on the CNN to solve this problem.

### 3.2 Photoelastic Image Dataset

It is expensive to obtain enough photoelastic fringe patterns and corresponding stress field images through the photoelastic experiment, which is due to the complicated experimental environment configuration and tedious post-data processing. Briñez-de León et al. (2020d) propose a hybrid scheme that includes real experimental data and computational simulation. Different types of light sources, the range of loading external forces, rotation angles of optical elements, and various types of camera sensors are fully considered in this method. According to a variety of different experimental conditions, such a rich isochromatic art dataset (Briñez-de León et al., 2020e) was finally synthesized through calculation methods. In this repository, all the experimental cases consider a PMMA material of 10 mm thickness and a stress optical coefficient of about 4.5 e^−12^ m^2^/N. There are totally 101,430 photoelastic image pairs in the dataset, and each pair includes the color fringe pattern and the corresponding gray stress map. The images are all 224 × 224 in size, and these images cover various patterns from simple to complex, which can be divided into two types: complete and patch. The fringe pattern and stress pattern are placed in different folders and matched by the same serial number.

### 3.3 Network Model

**FIGURE 2:**
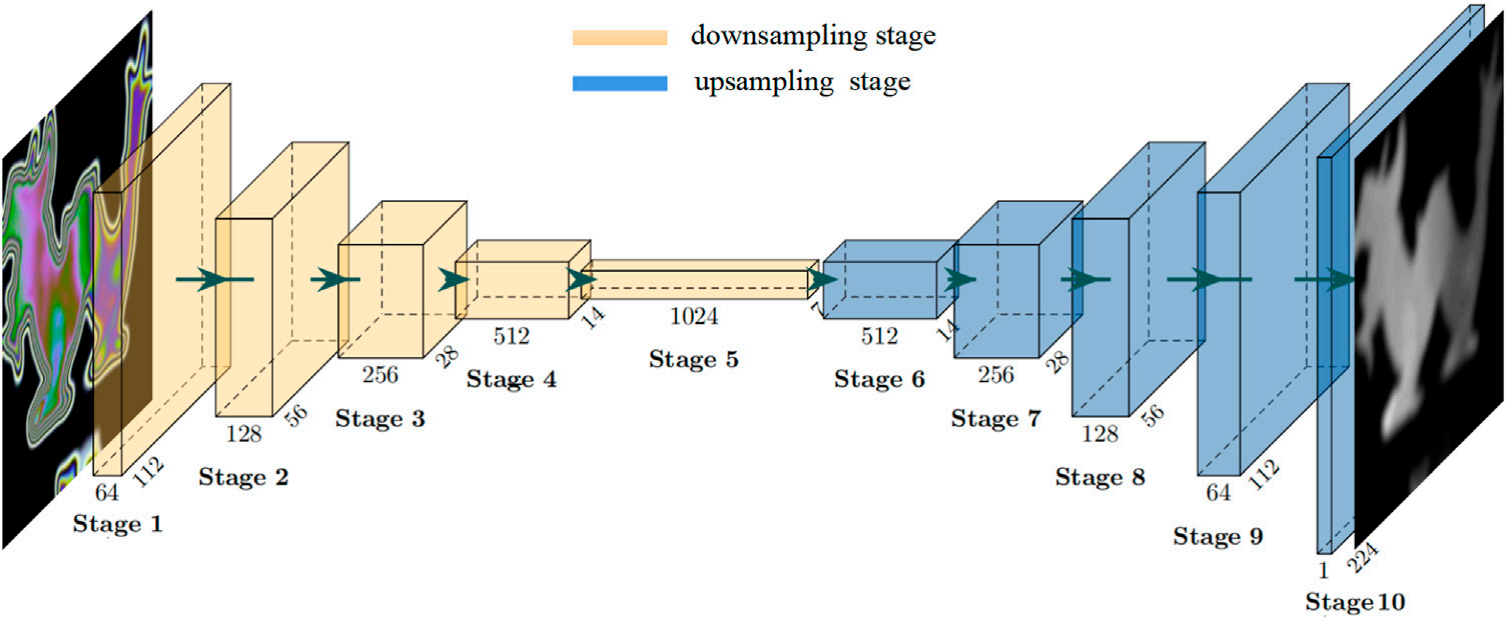
Schematic for the neural network architecture of the proposed model in this research. The network consists of an encoder and a decoder. An RGB isochromatic image is taken as the input, and the output is a stress map in gray scale.

We propose a stress field recovery model based on the encoder–decoder structure. The input of the network is a single photoelastic color fringe pattern, and the output is the gray image of the stress field recovered from the fringe pattern. The overall structure of the neural network model is shown in Figure 2.

**FIGURE 3:**
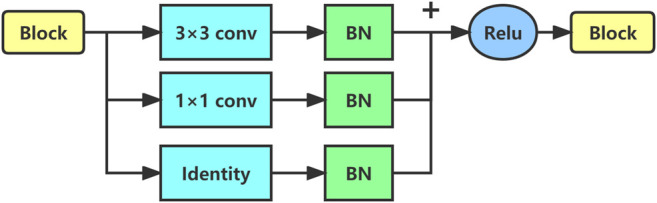
Sketch of RepVGGBlock architecture.

The whole network consists of an encoder and a decoder. The encoder receives the input photoelastic fringe pattern and then uses a series of sub-sampling convolution, batch normalization (BN) (Ioffe and Szegedy, 2015), activation, and other operations to complete feature extraction from the input images. The feature information extracted by the encoder will be used as an intermediate representation of the input fringe pattern. Concretely, the whole coding process is divided into five stages by using the structure of the cascaded convolutional neural network (Sun et al., 2020b; Weng et al., 2021), and each stage comprises a different number of RepVGGBlock (Ding et al., 2021), as shown in Figure 3. The number of blocks can be freely adjusted to change the encoder’s representation ability of the input image. Considering the trade-off between the accuracy and speed of the model, we set the number of blocks in each stage as {1,2,4,8,1}, and the image resolution of the first stage is 224 × 224. Experiments show that under the large image size, using only one block is helpful to speed up the training and reasoning of the model. The input and output dimensional parameters of each stage are shown in Table 1.

**TABLE 1:** Encoder parameters of each stage. The first parameter of the size indicates the number of channels, and the last two parameters indicate the size of the image during the down-sampling process.

Stage	Blocks	Input size	Output size
1	1	3 × 224 × 224	64 × 112 × 112
2	2	64 × 112 × 112	128 × 56 × 56
3	4	128 × 56 × 56	256 × 28 × 28
4	8	256 × 28 × 28	512 × 14 × 14
5	1	512 × 14 × 14	1024 × 7 × 7

RepVGGBlock is a convolutional block with multibranch topology, including convolution with 3 × 3 kernel branch, convolution with 1 × 1 kernel branch, and identity branch. Each branch performs BN operation, then concatenates them together, and finally outputs after being activated by ReLu. The schematic diagram of RepVGGBlock is shown in Figure 3.

**FIGURE 4:**
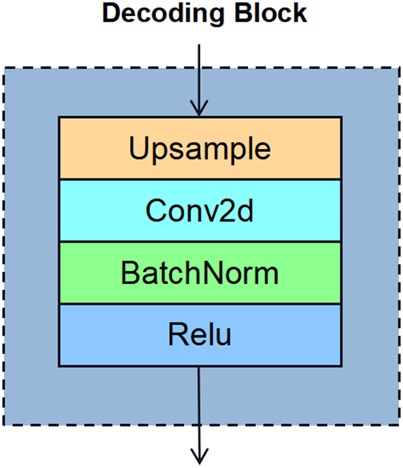
Sketch of decoding block architecture.

In order to recover the stress field distribution quickly and accurately, we designed a convolutional decoding structure which maps the features extracted by the encoder to the stress distribution map corresponding to the fringe pattern. The feature decoding process is also divided into five stages, each of which is stacked with blocks with the same structure. The number of blocks is also adjustable, and its structure comprises an up-sampling layer, a convolution layer, a BN layer, and an active layer. The schematic diagram is shown in Figure 4. The up-sampling layer uses bicubic interpolation (Huang and Cao, 2020), the convolution layer uses 3 × 3 conv, and the activation layer uses ReLu. Table 2 shows the parameters of each stage of the decoder.

**TABLE 2:** Parameters of each stage of the decoder. The input size of the decoder is the output size of the encoder, and the final output size of the model is 1 × 224 × 224.

Stage	Blocks	Input size	Output size
6	1	1024 × 7 × 7	512 × 14 × 14
7	2	512 × 14 × 14	256 × 28 × 28
8	2	256 × 28 × 28	128 × 56 × 56
9	2	128 × 56 × 56	64 × 112 × 112
10	1	64 × 112 × 112	1 × 224 × 224

### 3.4 Objective Optimization Function

Mean squared error (MSE) (choi et al., 2009; Ma et al., 2020) is the most commonly used loss function in image reconstruction, which has fast convergence speed. But for regression tasks, the MSE is prone to interference by outliers during training. Most importantly, it usually leads to blurred images (Gondal et al., 2018) because minimizing MSE is equivalent to minimizing the cross-entropy of the empirical distribution and Gaussian distribution on the training set. We followed the method proposed by Zhao et al. (2016) and used *L*1 (Liao et al., 2021), *L*2, and SSIM (Wang et al., 2004) to construct our stress recovery loss function. First, the *L*1 loss of the two images is defined as follows:

$${ℒ}^{L1}\left(I_{r},I_{g}\right)=\frac{1}{N}\sum_{p\in I}\left|I_{r}\left(p\right)-I_{g}\left(p\right)\right|,$$ (6)

where *p* is the index of the pixel; *I*_*r*_(*p*) and *I*_*g*_(*p*) are the values of the pixels in the recovered stress map and the ground truth, respectively; and *N* is the number of pixels *p* in the image *I*. Similarly, *L*2 loss is defined as follows:

$${ℒ}^{L2}\left(I_{r},I_{g}\right)=\frac{1}{N}\sum_{p\in I}{\left[I_{r}\left(p\right)-I_{g}\left(p\right)\right]}^{2}.$$ (7)

The SSIM has been widely used as a metric to evaluate image processing algorithms. It is a full reference image quality evaluation index which measures the image similarity from three aspects: brightness, contrast, and structure. The SSIM for pixel *p* is defined as follows:

$$\text{SSIM}\left(p\right)=\frac{2\mu_{x}\mu_{y}+C_{1}}{\mu_{x}^{2}+\mu_{y}^{2}+C_{1}}\cdot \frac{2\sigma_{xy}+C_{2}}{\sigma_{x}^{2}+\sigma_{y}^{2}+C_{2}}=l\left(p\right)\cdot cs\left(p\right),$$ (8)

where *x* and *y* are two image patches extracted from the same spatial position of the two images, respectively; *μ*_*x*_ and *μ*_*y*_ are the average brightness of patches *x* and *y*, respectively; *σ*_*x*_ and *σ*_*y*_ are the standard deviation of *x* and *y*, respectively; *σ*_*xy*_ is the covariance of *x* and *y*; and *C*_1_ and *C*_2_ are very small constants to avoid having a zero denominator.

In order to solve the problem of edge noise in the process of image generation, multiscale SSIM (MS-SSIM) is added to the loss function (Wang et al., 2003), which can effectively improve the impact of edge noise. MS-SSIM is defined as Eq. 7. M scale images were obtained by down-sampling. These images were evaluated by the SSIM, and the MS-SSIM value was obtained by fusion calculation.

$$\text{MS}\_\text{SSIM}\left(p\right)=l_{M}\left(p\right)\cdot \overset{M}{\underset{j=1}{\prod }}cs_{j}\left(p\right),$$ (9)

where *l*_M_ and *cs*_j_ are the terms defined in Eq. 8 at scales *M* and *j*. According to the convolutional nature of the network, the loss function of MS-SSIM can be written as follows:

$${ℒ}^{MS\_SSIM}\left(P\right)=1-MS\_SSIM\left(\tilde{p}\right),$$ (10)

where $\tilde{p}$ is the center pixel of patch *P* and *P* is the image patch sampled from image *I*_*r*_ and *I*_*g*_.

Finally, the objective optimization function is formulated in Eq. (11):

$$ℒ=\text{α}{ℒ}^{MS\_SSIM}\left(I_{r},I_{g}\right)+\beta {ℒ}^{L1}\left(I_{r},I_{g}\right)+\left(1-\alpha -\beta \right){ℒ}^{L2}\left(I_{r},I_{g}\right),$$ (11)

where *I*_*r*_ and *I*_g_ are the recovered stress map and the ground truth, respectively. Through the comparative experiments of different loss functions, we set α=0.5 and β=0.25 so that the different components of the loss function achieve roughly similar contributions.

## 4 Experiment and Analysis

By comparing the performance of the proposed network structure with the multiloss function fusion method on a synthetic dataset and comparing with the previous work, it is proved that the proposed method can accurately recover the stress field distribution from the photoelastic fringe pattern.

### 4.1 Implementation Details

Our network model is implemented by PyTorch. During the training process, 20,000 image pairs are randomly selected from the complete dataset, with 80% as the training set and the remaining 20% as the validation set. The batch size is set to 32, and Adam optimizer is used to train 100 epochs. The initial learning rate is 0.0001. The size of the input and output images is 224 × 224, in which the input is the RGB channel color fringe pattern and the output is the single channel stress gray pattern. We train the stress recovery network from scratch on a single NVIDIA GTX 1080Ti, save several models with low loss values on the verification set, and then select the one with the best performance on the validation set as the final model. Another 20,000 pairs of images are randomly selected as the test set, and these images did not appear in the previous training and validation sets. This test set will be used to evaluate the performance of the final model.

### 4.2 Comparison With Different Methods

We test the trained models on different number of test sets, and the test images are randomly selected from the data sets that have never participated in the training. The distribution map of the stress field recovered by the network is compared with the real distribution map. MSE, peak signal-to-noise ratio (PSNR) (Gupta et al., 2011), and SSIM are used to measure the quality of the generated stress field images and then compare with the previous work. The PSNR is an image quality reference value for measuring maximum signal and background noise, as shown in Eq. (12).

$$PSNR=10\log_{10}\left(\frac{MAX_{I}^{2}}{MSE}\right),$$ (12)

where *MAX*_I_ is the maximum possible pixel value of the image, and *MSE* is the mean square error.

For MSE, the value is close to 0 and the smaller the better. For the PSNR, high values indicate better performance; on the contrary, low values indicate low performance. For the SSIM, the value close to 1 indicates high similarity and the value close to 0 indicates low similarity.

The results of StressNet (Briñez de León et al., 2020b), GAN (Briñez de León et al., 2020c) and our proposed network are compared, as shown in Table 3. The experimental results show that the proposed network model has good feature extraction ability and better image space mapping ability. The large standard deviation of the PSNR and MSE may be due to the noise in randomly selected data, which leads to a certain deviation from the mean value. Generally speaking, the results obtained by our proposed stress recovery network are better than those of the previous work.

**TABLE 3:** Quantitative results. Comparison with StressNet (Briñez de León et al., 2020b) and GAN (Briñez de León et al., 2020c) in the SSIM, PSNR, and MSE.

Methods	SSIM		PSNR		MSE	
	Mean	SD	Mean	SD	Mean	SD
StressNet 1000 images	0.9587	0.0327	39.29	3.47	24.12	1.63
**Ours** 1000 images	**0.9925**	**0.0037**	**45.16**	7.20	**7.67**	17.14
StressNet 10000 images	0.9603	0.0329	39.33	3.58	25.18	1.47
**Ours** 10000 images	**0.9929**	**0.0041**	**44.96**	7.23	**7.88**	16.72
GAN 20000 images	0.9308	0.0186	28.36	1.67	1.36	0.04
Ours 20000 images	**0.9943**	**0.0043**	**44.99**	7.24	7.88	17.75

**FIGURE 5:**
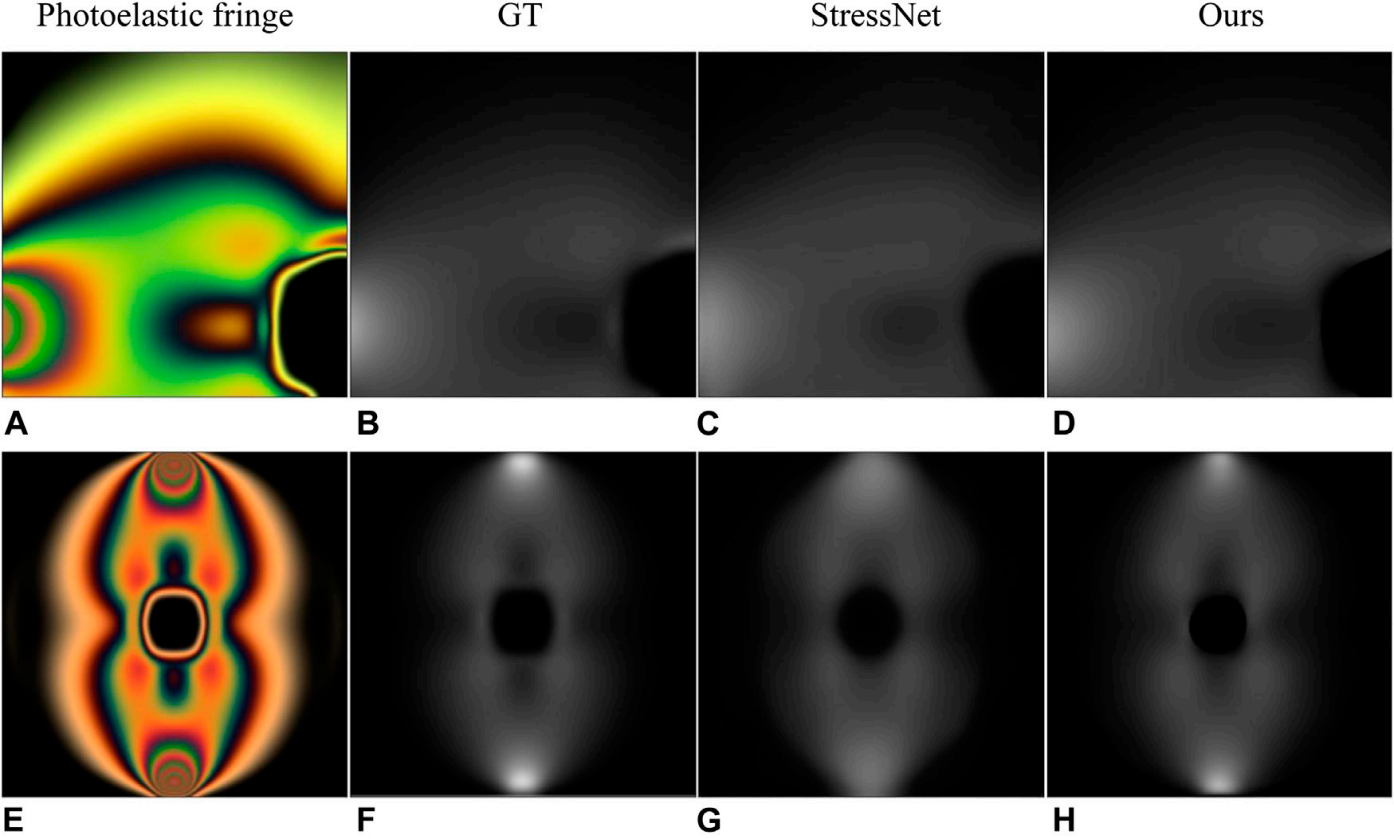
Results of comparison with StressNet (Briñez de León et al., 2020b). **(A,E)** are photoelastic fringes obtained with fluorescent and incandescent light sources, respectively. **(B,F)** are the corresponding ground truth. **(C,G)** are the results in StressNet. **(D,H)** are the results of our method.

We selected the two examples in StressNet to obtain specific instructions. The results in Figure 5 and Table 4 show that our proposed method has a high structural similarity index, and the bright area in the Figure 5 shows the degree of stress concentration. In order to measure the difference of the maximum stress difference between the recovery stress map and ground truth in the stress concentration area, the ratio of the maximum stress difference (RMSD) was proposed, as shown in Eq. 13. For the RMSD, the value close to 1 represents a small error. According to the RMSD value in Table 4, the stress recovery error of our method is smaller.

$$RMSD=\frac{MSD_{r}}{MSD_{g}},$$ (13)

where *MSD*_*r*_ and *MSD*_*g*_ are the maximum stress difference of the recovery stress map and ground truth, respectively.

**TABLE 4:** Quantitative results of comparison with StressNet (Briñez de León et al., 2020b). (a) and (e) represent the results of processing the photoelastic fringes in Figure 5.

Method	SSIM	PSNR	MSE	RMSD
StessNet(a)	0.6338	34.38	28.16	0.88
Ours(a)	**0.9916**	**40.61**	**5.85**	**0.89**
StessNet(e)	0.8476	34.27	75.86	0.59
Ours(e)	**0.9482**	**39.13**	**12.87**	**0.83**

**FIGURE 6:**
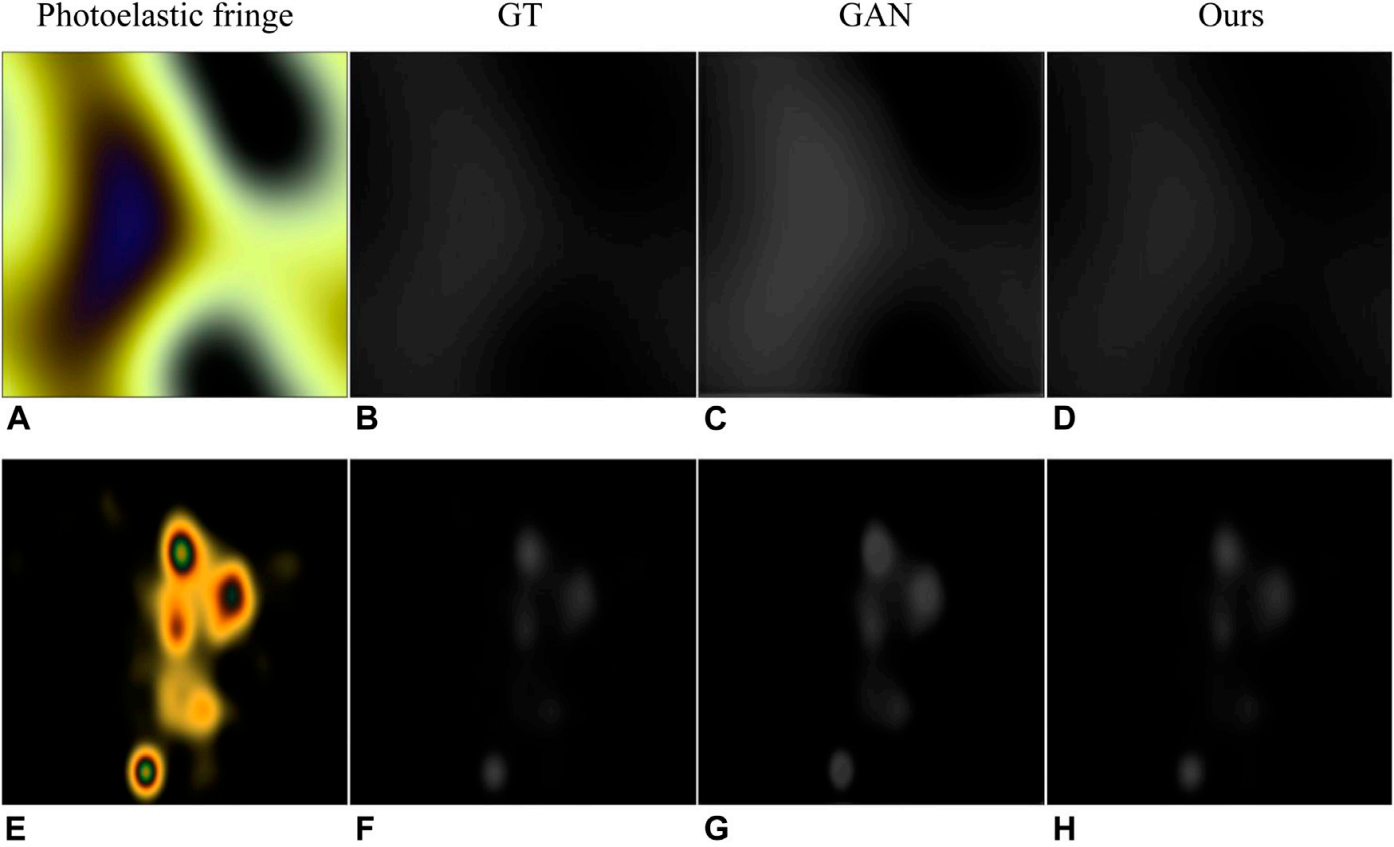
Results of comparison with GAN (Briñez de León et al., 2020c). **(A,E)** are photoelastic fringes obtained with Willard_LED and incandescent light sources, respectively. **(B,F)** are corresponding ground truth. **(C,G)** are the results in the GAN. **(D,H)** are the results of our method.

Similarly, we also make a further comparison with the methods proposed in GAN (Briñez de León et al., 2020c), as shown in Figure 6. The experimental results show that our method solves the local over-saturation phenomenon in GAN (i.e., the predicted value of the stress concentration area tends to be larger).

### 4.3 Comparison With Different Loss Functions

**FIGURE 7:**
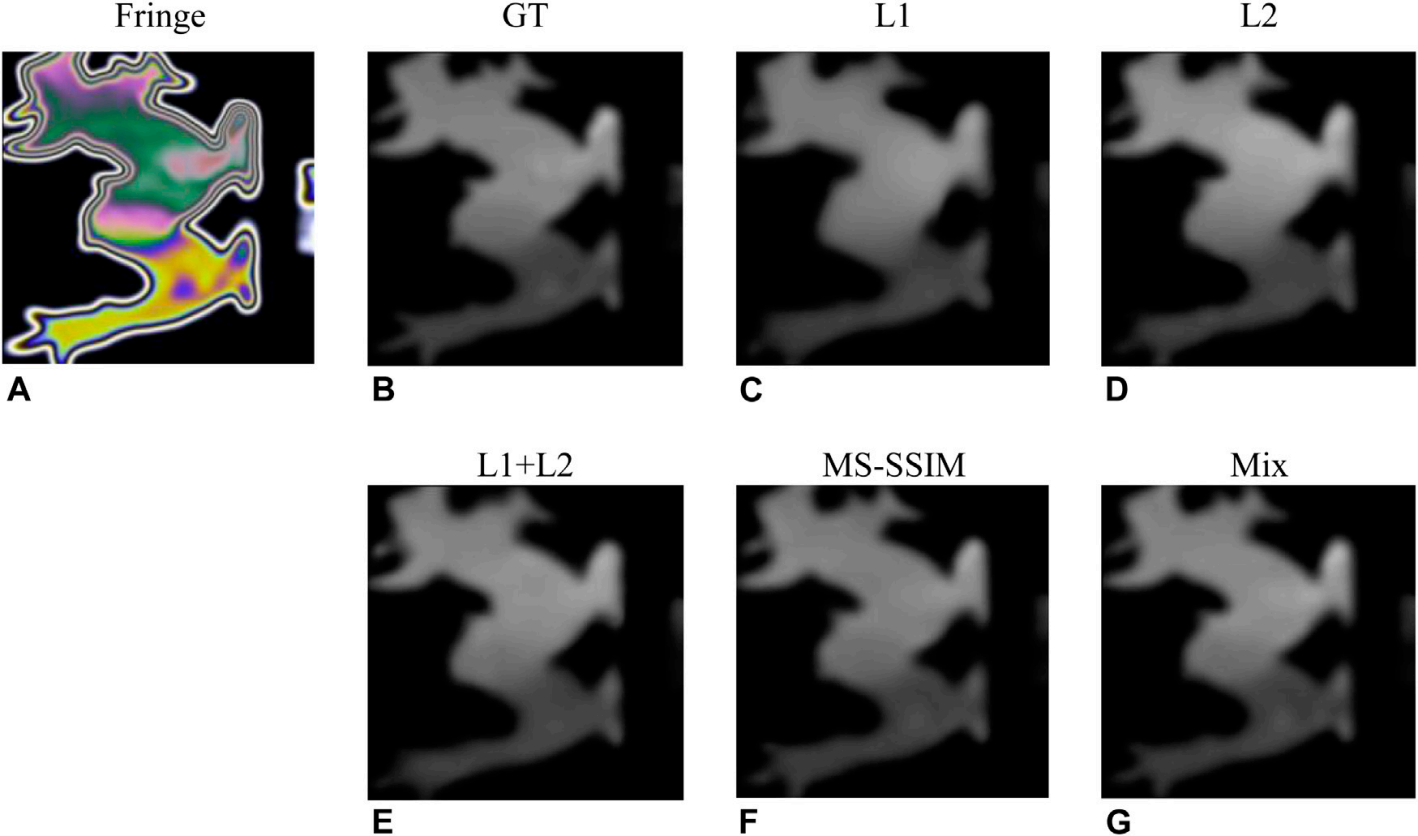
Results for different loss functions. **(A,B)** are image pairs randomly selected from the test set (Briñez-de León et al., 2020e) and **(C–G)** are the stress maps recovered under different loss functions.

In order to construct the most effective loss function to accurately recover the stress map, we compare the image recovery effect of the models trained under different loss functions. 20,000 images were used to evaluate each indicator to avoid accidental errors. The experimental conditions were the same except for different loss functions. The experimental results of each evaluation index are shown in Tables 5, 6. The results show that compared with using MSE and MS-SSIM as loss functions alone, the fusion of multiple loss function mix as shown in Eq. 11 can achieve better image recovery quality. Figure 7 shows a concrete example of stress map recovery under different loss functions. When L1, L2, and L1+L2 are used as loss functions, as shown in Figures 7C–E, local details are lost in the stress map recovery, leading to inaccurate results. When MS-SSIM is used as a loss function, as shown in Figure 7F, complete local details are preserved, but the maximum stress difference in the stress concentration area is less than the true value. Using the fusion loss function mix, as shown in Figure 7G, the best effect is achieved in all evaluation indicators, which is almost consistent with the ground truth.

**TABLE 5:** Quantitative results of comparison with GAN (Briñez de León et al., 2020c). (a) and (e) represent the results of processing the photoelastic fringes in Figure 6. MSE is calculated after image normalization.

Method	SSIM	PSNR	MSE	RMSD
GAN(A)	0.9144	25.201	30.186e-4	2.28
GAN(A)	**0.9974**	**50.148**	**1.02e-5**	**0.98**
GAN(E)	0.9390	35.196	3.022e-4	1.01
GAN(E)	**0.9923**	**52.497**	**1.10e-5**	0.96

**TABLE 6:** Comparison of results of different loss functions. All the results are tested on 20000 images, and then the average and standard deviation are obtained. MSE is calculated after image normalization.

Loss function	SSIM		PSNR		MSE		RMSD	
	Mean	SD	Mean	SD	Mean	SD	Mean	SD
L1	0.9803	0.0075	42.50	7.59	2.74e-4	7.16e-4	1.0276	0.0779
L2	0.9759	0.0164	43.36	**4.97**	1.02e-4	2.78e-4	1.0244	0.0985
L1+L2	0.9861	0.0101	43.80	5.09	**9.50e-5**	**2.59e-4**	1.0145	0.0879
MS-SSIM	0.9922	0.0057	43.08	8.35	2.88e-4	7.25e-4	1.0342	0.0852
Mix	**0.9943**	**0.0043**	**44.99**	7.24	1.43e-4	3.67e-4	**1.0109**	**0.0652**

### 4.4 Further Result Analysis

The prediction of the stress concentration area is the key point of practical engineering because it has a great influence on the fatigue life of components. We compared the maximum stress difference between the predicted stress map and ground truth and calculated the ratio of the maximum stress difference among 20,000 randomly selected image pairs.

**FIGURE 8:**
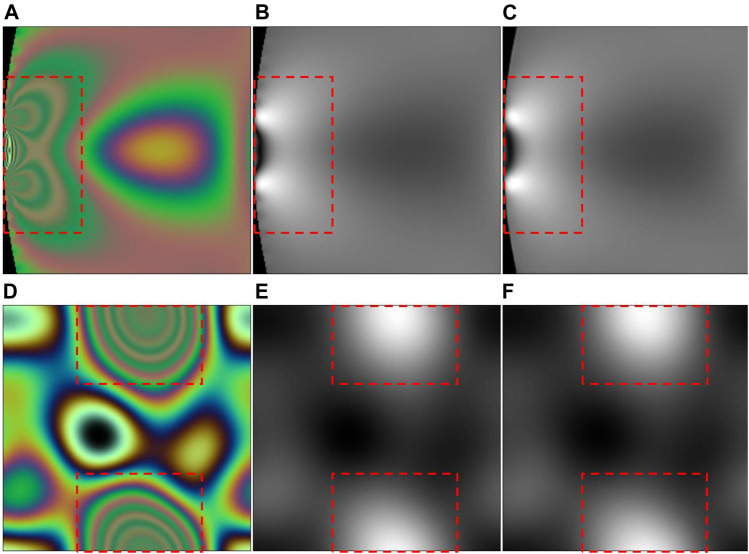
Stress concentration zones. Photoelastic fringes **(A,D)** and corresponding stress maps **(B,E)** are selected from the test set (Briñez-de León et al., 2020e). **(C,F)** are stress maps recovered by our proposed method. The region inside the red box is the stress concentration zone.

**FIGURE 9:**
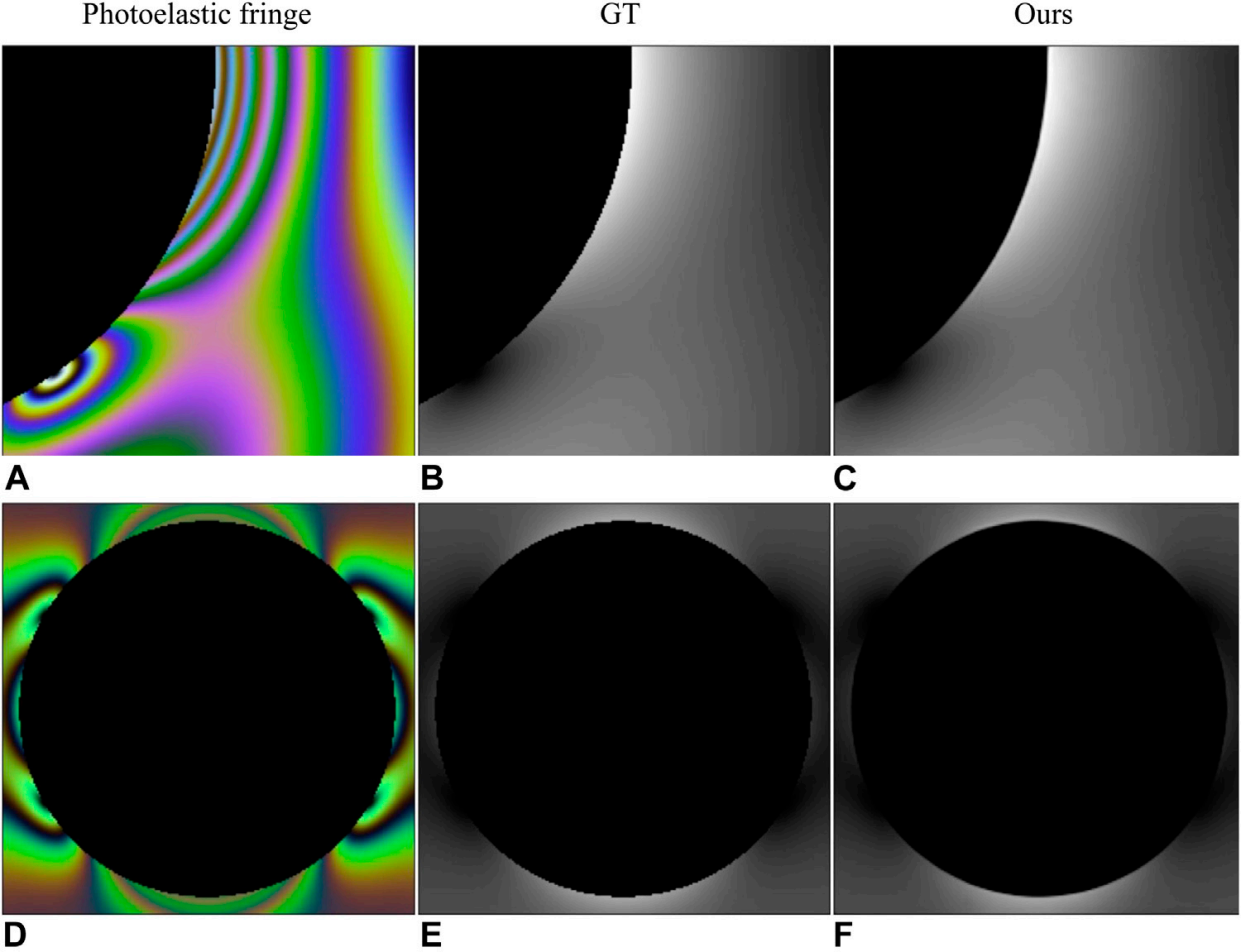
Edge of the stress map. Photoelastic fringes **(A,D)** and corresponding stress maps **(B,E)** are selected from the test set (Briñez-de León et al., 2020e). **(C,F)** are stress maps recovered by the proposed method. **(A)** is obtained by the human vision camera sensor with a cold white laser as the light source, and the maximum stress value of **(B)** is 72 MPa. **(D)** is obtained by a Sony_IMX 250 camera sensor with Willard_LED as the light source, and the maximum stress value of (e) is 48 MPa.

The experimental results show that the average RMSD value of 20,000 predicted and real stress maps is 1.0109, and the standard deviation is 0.0652. This indicates that our network model accurately predicts the maximum stress difference in the stress concentration area with very dense fringes, which is the benefit brought by the fusion of MS-SSIM and L1. Multiloss function fusion not only considers the change of global value but also has a good effect on optimizing the local minimum value in the training process. Figures 8, 9 list some specific examples. The color fringe images in Figure 8 are obtained by using a Sony_IMX250 camera sensor with a constant light source, and its maximum stress value is 72 MPa. Table 7 shows all the metric values of the recovered stress maps in Figure 8. The SSIM values of Figures 8C,F both exceed 0.99, and the maximum stress difference of them are 68.5 and 73.5 MPa, respectively. The results show that the maximum principal stress difference appears in the area with dense fringes, and their maximum stress difference error is less than 5%.

**TABLE 7:** All the metric values of the recovered stress maps in Figure 8.

Method	SSIM	PSNR	MSE	RMSD
(C)	0.9962	27.34	1.84e-4	0.9514
(F)	0.9957	35.24	3.02e-4	1.0222

In order to evaluate the performance of the proposed method at image edges, Figure 9 shows that the predicted stress map is very smooth in the edge area of the pattern, and the SSIM between the estimated stress map and ground truth in the edge area can reach 0.99. From the perspective of human vision, the prediction of stress concentration zones and edges is almost consistent with the ground truth. Table 8 shows all the metric values of the recovered stress maps in Figure 9.

**TABLE 8:** All the metric values of the recovered stress maps in Figure 9.

Method	SSIM	PSNR	MSE	RMSD
(C)	0.9939	33.11	4.92e-4	0.9885
(F)	0.9875	35.57	2.85e-4	0.9942

**FIGURE 10:**
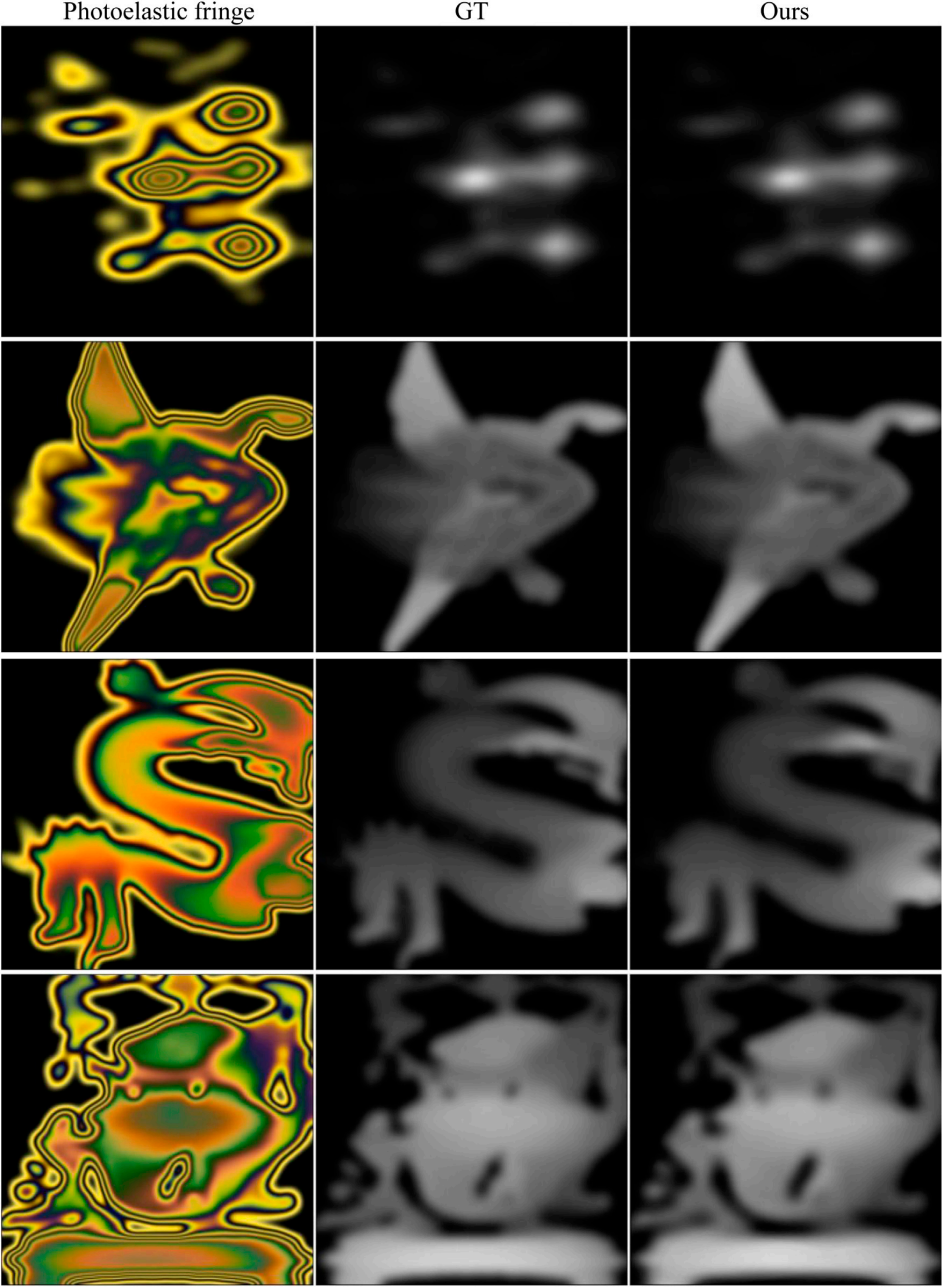
Stress recovery results of complex fringe patterns. First column: photoelastic color fringe pattern; second column: ground truth; third column: predicted stress map. All fringe patterns and corresponding stress maps are selected from the test set (Briñez-de León et al., 2020e). The maximum stress values of the ground truth from top to bottom are 72, 48, 48, and 60 MPa.

When solving the stress field of complex geometric objects under different experimental conditions, there were some problems in the previous study, such as complicated calculation methods and inaccurate calculation results. Figure 10 shows the advantage of our method in solving the stress field of complex geometry. Due to the strong unwrapping ability of the network model, the stress field recovery of the complex fringe pattern performs well in the edge and stress concentration area. The predicted results of the stress map in the first two rows in Figure 10 can reach 0.99 on the SSIM, and the more complex patterns in the last two rows can also exceed 0.97. Table 9 shows all the metric values of the recovered stress maps in Figure 10.

**TABLE 9:** All the metric values of the recovered stress maps in Figure 10.

Method	SSIM	PSNR	MSE	RMSD
(1)	0.9948	44.36	4.02e-5	0.9352
(2)	0.9903	38.35	1.51e-4	1.1121
(3)	0.9695	32.75	5.38e-4	1.0268
(4)	0.9583	30.49	8.94e-4	0.9543

### 4.5 Experimental Cases

**FIGURE 11:**
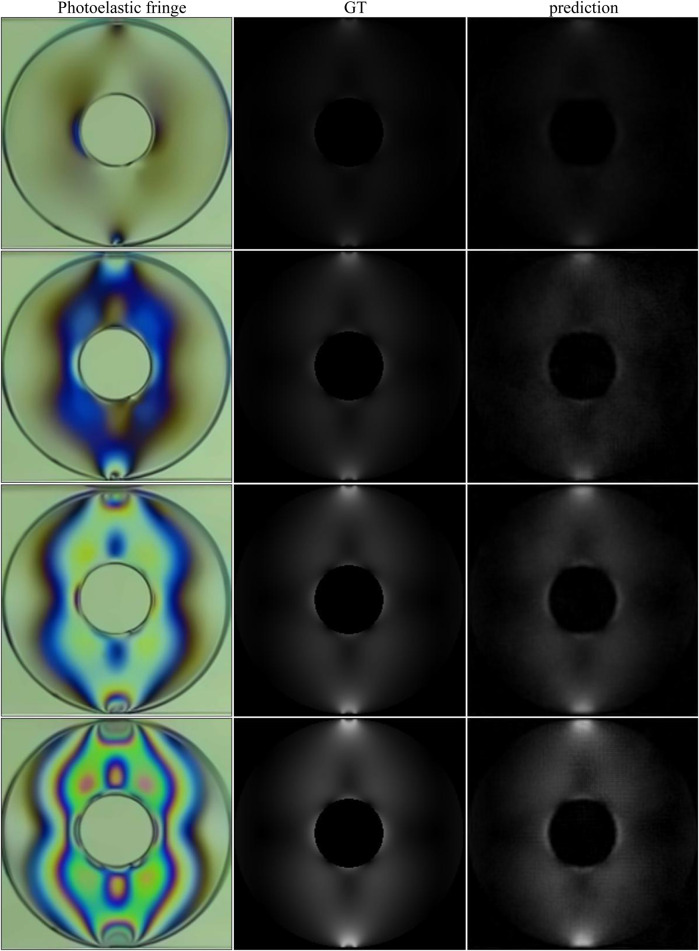
Stress recovery results of experimental cases. First column: photoelastic color fringe pattern; second column: ground truth generated by simulation; third column: predicted stress map.

The model was also evaluated when dealing with experimental cases. Based on experiments in literature (Restrepo Martínez and Branch Bedoya, 2020), 12 images were selected to fine-tune the network, and the remaining four images were used to test the performance of the network model. In the experiment, an LED was used as the light source, different loads were applied in a circularly polarized light field, and the photoelastic images were captured by the camera sensor DCC3260. The ground truth is generated by simulation. The test results are shown in Figure 11 and Table 10.

**TABLE 10:** All the metric values of the recovered stress maps in Figure 11.

Method	SSIM	PSNR	MSE	RMSD
(1)	0.9564	44.75	3.01e-5	0.9784
(2)	0.8758	37.55	1.80e-4	1.0171
(3)	0.9264	37.74	1.75e-4	0.9778
(4)	0.8796	35.34	2.92e-4	1.0263

## 5 Conclusion

We propose a deep convolutional neural network based on the encoder–decoder structure and design an objective optimization function weighted with multiple loss functions to recover the stress field distribution from color photoelastic fringe patterns. Verification results on open data sets show that our stress field recovery model can achieve an average performance of 0.99 on the SSIM. Other indexes also show that the model has the ability to accurately recover the stress map. When testing the photoelastic fringe patterns with complex geometry under different experimental conditions, our model still shows excellent generalization performance and strong unwrapping ability.

However, the proposed method only calculates the difference of principal stress. In practice, we also want to know the specific component of principal stress. In the future, a dataset containing principal stress components will be built, and then the principal stress can be obtained through the deep convolutional neural network.

## Data Availability

The original contributions presented in the study are included in the article/Supplementary Material, further inquiries can be directed to the corresponding authors.
